# THE JOINT EFFECTS OF PAIN AND ELEVATED DEPRESSIVE SYMPTOMS AMONG MEXICAN AMERICANS AGED 80 AND OLDER

**DOI:** 10.1093/geroni/igad104.2718

**Published:** 2023-12-21

**Authors:** Claudia Sanchez, Phillip Cantu, Kyriakos Markides, Sadaf Milani

**Affiliations:** University of Texas Medical Branch, Galveston, Texas, United States; UTMB, Galveston, Texas, United States; University of Texas Medical Branch, Galveston, Texas, United States; University of Texas Medical Branch, Galveston, Texas, United States

## Abstract

Pain and depression are common among older Hispanic adults and their combined effects may increase mortality. We examined the joint effects of pain and depressive symptoms on mortality. We used data from the Hispanic Established Population for the Epidemiologic Study of the Elderly (2010-2016), which included Mexican Americans aged 80 and older in the Southwestern US. Participants were categorized into four groups based on self-reported pain on weight-bearing and depressive symptoms: no pain or elevated depressive symptoms, pain only, elevated depressive symptoms only, or both pain and elevated depressive symptoms. Depressive symptoms were measured using the Center for Epidemiologic Studies Depression scale. A score of 16 or more was considered elevated depressive symptoms. Cox proportional hazards models were used to estimate the joint relationship of pain and depressive symptoms with mortality. We examined social support as a potential effect modifier. At baseline, 35.9% reported pain only, 8.6% of participants reported elevated depressive symptoms only, and 16.9% reported pain and elevated depressive symptoms (n=836). Over 6 years of follow-up, 46.2% of the sample died. Those with pain and elevated depressive symptoms had 1.41 times the risk of mortality compared to those without pain or elevated depressive symptoms (95% confidence interval: 1.05, 1.09). Having pain or elevated depressive symptoms only was not significantly associated with mortality. Social support was not a significant effect modifier. Our findings highlight the importance of screening for pain and elevated depressive symptoms among older Mexican Americans.

